# Supplementary material for: Neutralizing Antibodies in COVID-19 Patients and Vaccine Recipients after Two Doses of BNT162b2
Source: Viruses. 2021 Jul 14;13(7):1364. doi: 10.3390/v13071364 (PMC8309994; doi:10.3390/v13071364)
Supplement: Supplementary file 1 [file viruses-13-01364-s001.zip › viruses-1237233-SI.pdf]

Supplemental Material and methods: Pseudovirus neutralization test

(1) Samples

A total of 71 samples were analyzed by means of pVNT (23 pre-pandemic samples and 48 samples from COVID-19 patients).

(2) Material

- Living materials
  - HEK-293T modified to express human ACE receptor (hACE2) (Invivogen, San Diego, CA, USA).
  - SARS-CoV-2 Pseudoviral Particles are replication-deficient Maloneymurine Leukemia Virus (MLV) pseudotyped with the SARS-CoV-2 spike protein carrying the original D614 genotype (E-enzyme, Gaithersburg, MD, USA)
- Non-living materials
  - Sterile white 384-well cell culture plate with flat bottom to minimize luciferase-related inter-well reading interference (Greiner Bio-One, Kremsmünster, Austria).
  - Sterile 384-well plate with round bottom to allow maximum recovery of liquid during serial dilution steps (Waltham, MA, USA).
  - Dulbecco's Modified Eagle Medium (DMEM) with L-glutamine. This medium is used as a culture medium and as a dilution medium (Lonza, Bâle, Switzerland).
  - FireFly Luciferas kit is use to reconstitute the luciferas revelator which permit to dectect the infected cells (E-enzyme, Gaithersburg, MD, USA).

(3) Methods

- *On day 1:* A cell count is performed using trypan blue to ensure that 8,500 cells in 15 µL will be dispensed per well. The cells are then dispensed into all the necessary wells of the 384-well plate. The cells are then incubated for 24 hours in an incubator at 37°C and 5% CO_2.
- *On day 2:* The first dilution plate is fill with 20 µL of DMEM, in each first well of each sample, 20 µL of sample are added to the medium Serial dilutions of sera are then performed dilutions range from 1/2 to 1/128. Then, 7.1 µL from each well of this first plate is transferred in duplicate to the second dilution plate. The serum dilutions are incubated with 17.9 µL pseudovirus for 2 hours at 37°C and 5% CO_2.

Once the incubation is finished, 7.5 µL of the diluted serum + pseudovirus are put on the cells and 17.5µL of DMEM is added in each well. The plate is put to the incubation for 48 hours at 37°C and 5% CO_2. The final dilutions range from 1/10 to 1/640.

- *On day 3:* the final plate containing the cells with serum and pseudovirus is emptied of its liquid content and filled with 20 µL of fresh reconstituted luciferase reagent. The plate is then read in luminescence on the SpectraMax iD3.

In addition, a cell control and a viral control are performed in at least one plate. The cell control consists of a classical cell growth with culture medium. The data obtained are considered as background.

The viral control follows the same scheme as the samples except that pre-pandemic serum is used, the data obtained gives the value of 100% infectivity. The data obtained are expressed in relative light unit (RLU) and are inversely proportional to the presence of NAbs.

Indeed, luciferase contained in the virus genetic material allows to detect infected cells when the luciferase detection reagent is added. Luciferas will cleave the luciferin in the reagent which gives a luminescent signal.

The purpose of this manipulation is to determine the dilution at which 50% infectivity inhibition is obtained. Each dilution will give us a percentage of inhibition and the dilution at which we reach 50% is considered as the result of our test. Some samples have a very high neutralizing antibody titer, which makes it impossible to determine the dilution at which 50% of inhibition is obtained in the previously presented dilution range. It is therefore necessary to select these samples and run them in a second round of analysis. This second series of analysis is carried out according to the procedure described above with the exception of the dilution range, which is extended to 1/5,120.

- *Statistical Analysis pVNT*

Based on the RLU values of each sample, a percentage of inhibition can be calculated. The following formula must be applied to each dilution for each sample:

$$Relative\ inhibition = \frac{RLU\ sample\ X - RLU\ cell\ control}{RLU\ viral\ control - RLU\ cell\ control}$$

The different percentages of inhibition are used to plot the evolution of the relative inhibition as a function of the serum dilution. By intrapolation of the obtained sigmoïd curves, it is possible to determine the dilution for which 50% of inhibition is reached. A sample is considered as negative if the half maximal inhibitory concentration (IC_{50}) value of this sample is below the dilution 1/20.
